# Supplementary material for: Sex differences in the immediate extinction deficit and renewal of extinguished fear in rats
Source: PLoS One. 2022 Jun 10;17(6):e0264797. doi: 10.1371/journal.pone.0264797 (PMC9187087; doi:10.1371/journal.pone.0264797)
Supplement: Attachment — Submitted filename: PLOS_reviewer_comments_v1.1.docx [file pone.0264797.s002.docx]

*We would like to thank both reviewers for their thoughtful comments and suggestions. We believe that the current version of the manuscript has been significantly improved based on these suggestions.*

Reviewer #1: This manuscript examined whether or not biological sex has an effect on the retrieval of extinction memory when rats are exposed to extinction training within minutes of fear conditioning versus extinction training given 24 hours after conditioning. The authors also tested whether behavioral performance differed in males in females given a renewal test after extinction. The manuscript is well written and the experiments are well-designed. I have some comments related to analysis and interpretation that I thing might be useful to address

1) I wonder if the authors can speak to whether or not the deficit in extinction when it occurs shortly after conditioning might not be an effect on extinction per se, but instead reflects enhanced conditioning that is not evident on the early trials of extinction because performance is at ceiling.

**The conditioning procedures are identical for rats in the immediate and delayed extinction conditions. Conditioned freezing during the conditioning session and, as the reviewer points out, during the earliest trials of the extinction session are similar in the two conditions. Different levels of conditioning cannot account for the differential extinction retention shown after immediate or delayed extinction procedures.**

2) Experiments 1 and 2 appear to be analyzed differently with experiment 1 being analyzed with trials, extinction type, and sex as factors, while experiment 2 used blocks instead of trials. I wonder if the authors could explain why the difference.

**This was an error. Both experiments used 5-trial blocks in the analysis of the 45-trial extinction/retrieval sessions. This typo has been corrected in the current version of the manuscript.**

3) Since there was no context shift, the baseline periods of the extinction training session functions as a test of context fear. The authors should comment on the lack of a difference between males in females (which contradicts some prior work) and how it fits with their explanation of the sex difference in renewal as reflecting a difference in contextual processing. The authors might also comment on why there is such a large discrepancy in experiments 1 and 2 in contextual fear. Is this simply a strain effect?

**This is an excellent point, and we have now reported an additional analysis in the results and discussed this issue in the manuscript. The essential point is that in the first experiment the pre-CS period during the extinction session is confounded by nonassociative shock sensitization. However, in the second experiment, the Delay-No-Extinction controls experienced a context test unconfounded by sensitized fear. In this case, males exhibited significantly greater contextual freezing relative to females, as previously reported.**

**In terms of overall levels of contextual fear, indeed there is a robust strain difference in this dependent variable. We now discuss this issue in both the results and discussion. Namely, Wistar rats show very little freezing to conditioned contexts. This was also reported by the lab that originally developed this strain (Fig. 4, Pomrenze, et al. 2019; DOI:** [10.1016/j.celrep.2019.08.083](https://dx.doi.org/10.1016%2Fj.celrep.2019.08.083)). **In that paper, they show that this strain of Wistar rats only froze at ~20-40% during the first minute of context retrieval and quickly reduced to ~10% freezing during minutes 2-5. Here, we found that Wistar rats averaged < 20% during the 3-minute baseline in the conditioning context (Fig. 2) which is in agreeance with their findings.**

**We completely agree that these are important points of emphasis and have updated our Results and Discussion sections to include these. Thank you for your attention to detail!**

4) The conclusion that immediate extinction produces less long-term extinction is based on performance during the retrieval test. In both experiments 1 and 2, there is no difference during the first block of 5 trials of the retrieval test, but a difference emerges on the subsequent trials. This is reported as a trial (or block) by extinction type interaction. I wonder why this interaction is taken as evidence of a difference in extinction retention, while the lack of an effect on the first 5 trials is not evidence of a lack of an effect. Especially when considering how common it is in the literature for extinction retention to be assessed by analyzing the initial trials of retrieval, and not the later trials which would be confounded by re-extinction.

**Although we have reported that the IED can yield differences in the earliest trials of an extinction retention test, it is more commonly expressed as a faster rate of re-extinction in delay animals (see Chang and Maren, 2009, Learning and Memory). we infer that this enhanced savings in delay relative to immediately extinguished animals is due to an extinction learning impairment. We have updated the manuscript to make this clearer.**

Reviewer #2: The present manuscript describes two experiments aiming to examine potential sex differences in the immediate extinction deficit (IED), finding no sex-based differences in two strains of rats. A sub-experiment demonstrates that in Wistar rats, females fail to show AAB renewal following a complete IED experiment and re-extinction. Overall the paper is well-written and the experiments are appropriately conducted and analyzed, but the general conclusion that females fail to show renewal of a fear response is difficult to reconcile with the broader literature. Instead, this effect might be an artifact of the procedure employed here which involved different extinction treatments ahead of the renewal testing.

1) Renewal of a fear response has been well-documented in female Wistar rats. For example, the Bouton & Bolles (1979) demonstration of fear renewal used exclusively female Wistar rats. One procedural difference between the Bouton & Bolles results and the results reported here is the use of conditioned suppression in the 1979 demonstration and use of freezing here. It should be stated that renewal has also been observed in female Wistar rats when freezing was the dependent variable of interest (e.g., Harris & Westbrook, 1998, Expt. 5; Morris & Bouton, 2007). A failure to obtain fear renewal in the present study was therefore unlikely to be due to a difference in the dependent variable of choice and instead attributable to other factors here.

**We thank this reviewer for their insightful comments and directing us to consider this issue. This reviewer suggests that our findings that females failed to display fear renewal cannot be due to sex differences considering previous literature showing that females rats do show renewal. We now acknowledge in the discussion recent and historical literature that has found renewal in female rats. It is not clear what accounts for these differences across laboratories, but in our hands the greater contextual freezing in male rats might indicate that contexts have greater control over their behavior. Given that extinction is highly context-specific, this predicts greater renewal in male rats. As detailed below, an artifact of prior group assignments in the IED procedure does not account for our findings. We have reworked our discussion to make this clearer and incorporated the above references showing that female rats can indeed renew.**

2) Most notably, the authors used non-naïve rats who had previously undergone conditioning through an IED paradigm which involved Pavlovian fear conditioning, extinction, and testing. While the authors did report difference between immediate and delayed extinction procedures in later extinction recall, the groups are collapsed for the renewal experiment. I think it would be worth analyzing the data taking into account prior group assignment (immediate vs. delayed) to examine if differences in the formation of the original extinction memory are contributing to the effects observed in later renewal.

**We have re-analyzed this data using prior IED group assignments, including both Ext (Extinction vs No-Extinction) and Ext-type (Immediate vs Delayed) as variables, as suggested by this reviewer. We have included this analysis in the paper. We found that there was a significant Sex x Context x Trial interaction (*F*_(5, 120)_ = 2.96, *p* = .015) indicating a sex difference in renewal, but no significant interaction of Sex x Ext-type x Trial (*F*_(5, 120)_ = 0.920, *p* = .471), Context x Ext-type x Trial (*F*_(5, 120)_ = 1.531, *p* = .185), or Sex x Context x Ext-type x Trial (*F*_(5, 120)_ = 0.969, *p* = .440). There was a significant Context x Ext x Trial interaction (*F*_(5, 120)_ = 2.367, *p* = .044) driven by NoExt animals freezing more in Ctx A, and less in Ctx B, relative to Ext animals. However, Sex did not significantly interact with prior Ext assignment in any way [Sex x Ext x Trial (*F*_(5, 120)_ = 0.795, *p* = .556); Sex x Context x Ext x Trial (*F*_(5, 120)_ = 1.160, *p* = .333)]. In both of these analyses, male rats tested in the renewal context (B) showed higher freezing than all other groups during the first two trials, regardless of previous grouping in the IED procedure.**

**From this we can conclude that prior group assignment in the IED procedure cannot explain the lack of renewal we observed in Female rats. This is unsurprising as we counterbalanced renewal groups such that each renewal group contained equal number of animals from each of the prior IED groups. We have these statistics in the current version of the manuscript.**

3) An additional caveat to the present results is that rather than employing an ABA renewal design, the authors examined AAB renewal which is a relatively less robust effect. There are a few possible explanations of the results. Either, as the authors state, females are less likely to show AAB renewal than males (which is interesting in its own right as there are several examples of ABA renewal in females) or something about the prior experience with extinction in the IED paradigm renders females less susceptible to AAB renewal.

**ABA indeed results in higher levels of renewal, but this also conflates fear to the CS with fear to the conditioning context. We chose to use an AAB renewal procedure to avoid this confound, and our lab routinely observes renewal using both AAB and ABC designs. It is possible that female rats would be more likely to renew in an ABA design, however, the fact that males (and not females) exhibited AAB renewal suggests sex differences in fear renewal. As shown above, the previous grouping in the IED paradigm cannot explain these findings.**

4) I hesitate to suggest new experiments because I am acutely aware of the amount of work that goes into running these, but I don’t see how the authors can make concrete claims on the inability for females to demonstrate fear renewal without running additional experiments. I do think that in order to publish this with the current data, a new analysis including groups separated in the renewal test based on prior group assignment as well as discussions highlighting procedural differences here that might contribute to the lack of renewal, especially in the context of many published demonstrations of renewal in female Wistar rats, would be important.

**We deeply appreciate this reviewer’s awareness of the amount of effort it takes to run additional experiments. To alleviate the concerns expressed here without running additionally experiments we have: 1)** **ran the additional analyses suggested by this reviewer, 2) clarified our argument that there are situations/mechanisms by which female rats do not show renewal (as evidenced by us and others) but this does not mean that females are incapable of displaying renewal, and 3) added discussion around the previously overlooked literature and procedural differences within. We appreciate this review’s careful critiques and believe that these changes have improved the overall quality of our findings and accompanying manuscript.**
